# Carbon dioxide-enhanced angiography for detection of colonic diverticular bleeding and clinical outcomes

**DOI:** 10.1186/s42155-024-00481-3

**Published:** 2024-09-13

**Authors:** Ryoichi Kitamura, Takaaki Maruhashi, Reiko Woodhams, Koyo Suzuki, Yutaro Kurihara, Kaoru Fujii, Yasushi Asari

**Affiliations:** 1 Department of Emergency and Critical Care Medicine, Kitasato University School of Medicine, 1-15-1 Kitasato, Minami-Ku, Sagamihara, Kanagawa 252-0375 Japan; 2 Department of Comprehensive Medicine, Division of Interventional Radiology, Research and Development Center for New Medical Frontiers, Kitasato University School of Medicine, Kanagawa, Japan; 3 Department of Diagnostic Radiology, Kitasato University School of Medicine, Kanagawa, Japan

**Keywords:** Carbon dioxide angiography, CO_2_ angiography, Diverticular bleeding, Lower gastrointestinal bleeding, Transcatheter arterial embolization

## Abstract

**Purpose:**

To determine the ability of CO_2_-enhanced angiography to detect active diverticular bleeding that is not detected by iodinated contrast medium (ICM)-enhanced angiography and its impact on clinical outcomes when used to confirm embolization, particularly the risks of rebleeding and ischemic complications.

**Materials and methods:**

We retrospectively identified a cohort of patients with colonic diverticular bleeding who underwent catheter angiography between August 2008 and May 2023 at our institution. We divided them according to whether they underwent CO_2_ angiography following a negative ICM angiography study or to confirm hemostasis post-embolization (the CO_2_ angiography group) or ICM angiography alone in the absence of active bleeding or for confirmation of hemostasis post-embolization (the ICM angiography group). The ability to detect active colonic diverticular bleeding and clinical outcomes were compared between the two groups.

**Results:**

There were 31 patients in the ICM angiography group and 29 in the CO_2_ angiography group. The rate of detection of active bleeding by CO_2_ angiography that was not identified by ICM angiography was 48%. The rebleeding rate was 23% in the ICM angiography group and 6.9% in the CO_2_ angiography group. Among the patients who underwent TAE, the ischemic complications rate was 7.1% in the ICM angiography group and 4.5% in the CO_2_ angiography group.

**Conclusions:**

CO_2_ angiography may detect active diverticular bleeding that is not detectable by ICM angiography and appears to be associated with a lower rebleeding rate.

**Level of evidence:**

IV.

**Graphical Abstract:**

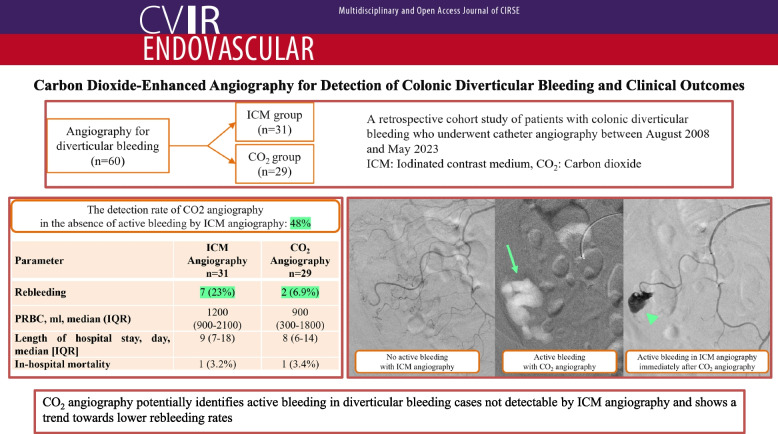

## Introduction

Diverticular disease is the most common cause of bleeding in the lower gastrointestinal tract and has a high incidence [1–3]. Rebleeding is reported to occur in around 40% of cases [4] and significantly affects quality of life for patients. [5] Therefore, there is an urgent need to reduce the risk of rebleeding. Transcatheter arterial embolization (TAE) has been shown to be effective for reducing the rate of rebleeding from the lower gastrointestinal tract, including diverticular bleeding, with several studies showing a reduction in bleeding risk by 17.4%–27.9% [6–9]. However, the active bleeding rate observed during catheter angiography remains at approximately 49% [6, 10]. Moreover, the intermittent nature of active diverticular bleeding may lead to difficulties in identifying the target vessels. In such cases, carbon dioxide (CO_2_)-enhanced or provocative angiography may be useful in terms of confirming the diagnosis [11, 12]. CO_2_ is more effective in detecting bleeding hydrodynamically than iodinated contrast agents [13]. Additionally, CO_2_ angiography has low viscosity and vasodilatory effects and has recently attracted attention as a useful method for diagnosis of active bleeding in the lower gastrointestinal tract, obstetric hemorrhage, and ruptured hepatocellular carcinoma when iodinated contrast medium (ICM)-enhanced angiography has failed [11, 14, 15]. However, CO_2_ angiography is not widely performed to detect the source of active bleeding in the lower gastrointestinal tract, and it remains unclear whether detecting and embolizing such sites with CO_2_ angiography has clinical benefits or if embolization contributes to adverse events.

The purpose of this study was to determine the ability of CO_2_ angiography to detect active diverticular bleeding and the outcomes of its use to confirm embolization, including the risks of rebleeding and ischemic complications.

## Materials and methods

### Study design and patient population

The study had a retrospective design and was approved by our institutional review board (approval number B23-028). The need for informed consent was waived. Patients with diverticular bleeding who had undergone catheter angiography between August 2008 and May 2023 were identified by a search of the medical records. Patients who were transferred to another hospital immediately following treatment and those for whom the medical records were incomplete were excluded.

### Data collection

The patients were divided into those who underwent CO_2_ angiography following a negative ICM angiography study or to confirm hemostasis post-embolization (the CO_2_ angiography group) or ICM angiography alone in the absence of active bleeding or for confirmation of hemostasis post-embolization (the ICM angiography group). Data on patient demographics, including age, sex, body mass index (calculated as kilograms of body weight divided by height in meters squared), comorbidities, medications), clinical manifestations (coagulopathy and hemodynamic instability), laboratory results, computed tomography (CT) findings (bleeding site and interval between CT and angiography), and angiographic findings (including details of CO_2_ angiography, embolization, and technical success). Information was also obtained on clinical outcomes, included rebleeding during hospitalization, need for massive transfusion, amount of transfusion required during hospitalization, length of hospitalization, and in-hospital mortality. Evaluation of embolization and major complications was limited to patients in whom embolization was performed. Rebleeding was also assessed in the embolization cohort.

### Definitions

Coagulopathy was defined as follows: a prolonged prothrombin time (international normalized ratio > 1.5); thrombocytopenia (platelet count < 80,000/ml); or a prolonged activated partial thromboplastin time (> 45 s) [16]. Hemodynamic instability was defined as hypotension (systolic pressure < 90 mmHg) and/or tachycardia (heart rate > 100 beats/min). Massive transfusion was defined as requirement for 1,500 ml or more of packed red blood cells.

Technical success was defined as complete cessation of blood flow in the angiographically targeted vessel. Technical failure was defined as inability to perform embolization owing to absence of active bleeding and no clear target vessel on ICM-enhanced or CO_2_-enhanced angiography, or because of vasospasm caused by the guidewire.

Rebleeding was defined as the presence of acute bleeding signs requiring immediate therapy (transfusion of 300 ml or more of packed red blood cells or an endovascular, endoscopic, or surgical intervention during the hospitalization period). Major ischemic complications were defined as follows: ischemia or infarction of the intestines requiring surgical or endoscopic treatment or fasting for more than 2 days. Asymptomatic ischemic changes observed incidentally during lower gastrointestinal endoscopy after embolization were not considered complications of the embolization procedure.

### Catheter angiography and embolization technique

**Fig. 1:**
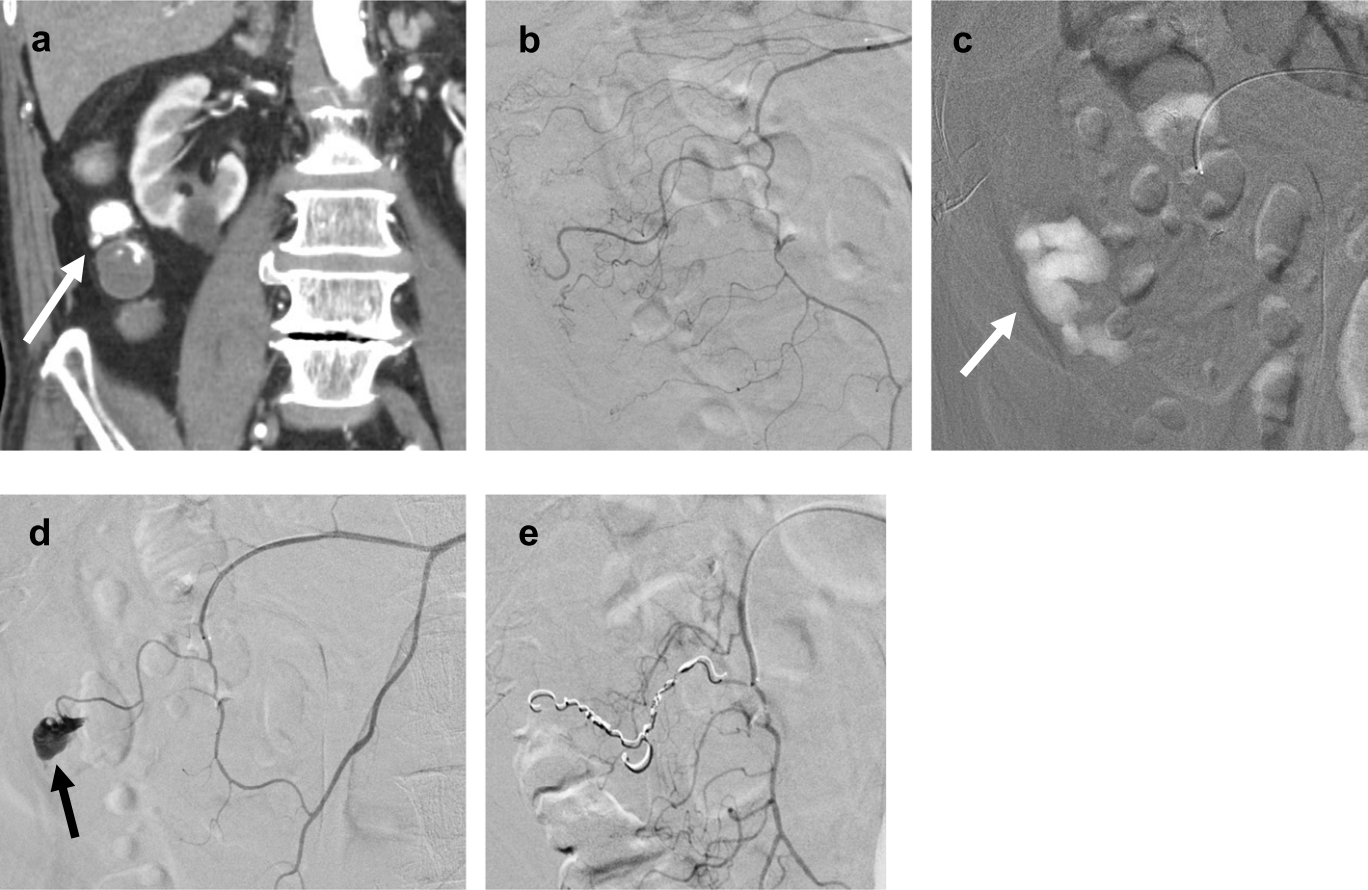
A male in his 80 s who underwent TAE for diverticular bleeding using CO_2_ angiography. (**a**) CECT revealed active bleeding from a diverticulum of the ascending colon (white arrow). (**b**) Angiogram from the marginal artery showed no active bleeding. (**c**) CO_2_ angiography from the marginal artery revealed the active bleeding in the vasa recta (white arrow). (**d**) After performing CO_2_ angiography, iodine contrast media-enhanced angiography was conducted immediately, confirming that active bleeding had been induced (arrow). (**e**) After embolization with microcoils, it was confirmed that hemostasis was complete

A 5-Fr vascular access sheath was inserted into the common femoral artery. A 0.035-inch hydrophilic guide wire (Radifocus; Terumo, Tokyo, Japan) and a modified 5-Fr shepherd hook-type catheter (Hanaco Medical, Saitama, Japan) were used to select the superior mesenteric artery (SMA) or inferior mesenteric artery for angiography. Using the conventional approach, if the bleeding source could be identified, a microcatheter (Progreat Lambda; Terumo) was inserted up to the vasa recta, and embolization was performed using microcoils (Galaxy, Johnson & Johnson, Tokyo, Japan; Target, Stryker, Tokyo, Japan; or Hilal, Cook Medical, Tokyo, Japan). According to the operator's discretion, imipenem/cilastatin), N-butyl cyanoacrylate (Histoacryl; B. Braun, Melsungen, Germany), or gelatin sponge (GS) (Serescue; Astellas Pharma, Tokyo, Japan) could also be used. When the source of bleeding could not be identified by angiography of the main branches of the SMA or inferior mesenteric artery, a microcatheter was super-selectively inserted according to whether the bleeding source was suspected to be the vasa recta on contrast-enhanced CT or located near the clips placed during endoscopy. Initially, an iodinated contrast agent is used for vascular imaging. Should the bleeding source remain elusive after super-selective angiography with ICM, the operator may opt for CO_2_ angiography. The procedure for CO_2_ angiography involves manually injecting CO_2_ from a 5-ml syringe into the microcatheter at a rate of approximately 2.5 ml per second under manual pressure. We perform CO2 angiography on the vasa recta or marginal artery near the diverticulum suspected by CT or endoscopy. It is also performed on the ileocolic artery, right colic artery, middle colic artery, sigmoid artery, left colic artery, or superior rectal artery. After CO_2_ angiography, ICM angiography is performed immediately without changing the position of the microcatheter. Observation is the course of action often taken even if active bleeding remains unclear after CO_2_ angiography. However, empirical embolization may be performed using coils or imipenem/cilastatin in cases with repeat recurrent bleeding (Fig. 1).

### Statistical analysis

All patient data were summarized using descriptive statistics. The data are presented as the median and interquartile range (IQR) or as the number and percentage.

## Results

**Fig. 2:**
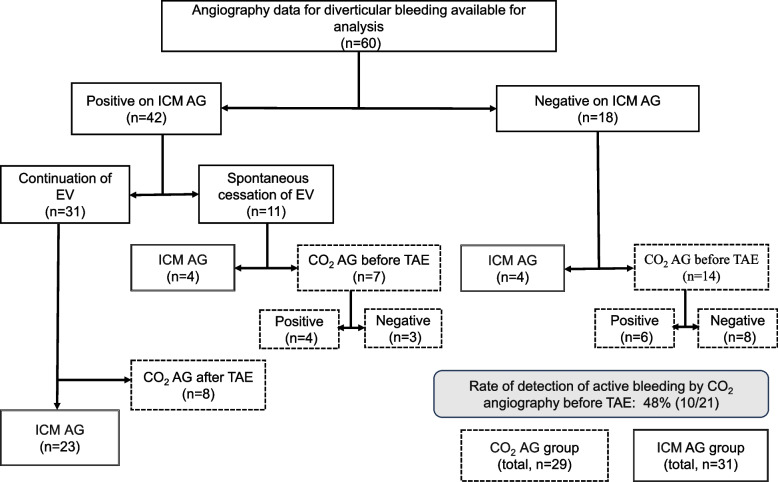
AG, angiography; EV, extravasation; ICM, iodinated contrast medium; TAE, transcatheter arterial embolization

Of 63 patients with diverticular bleeding who underwent catheter angiography at our hospital between August 2008 and May 2023, 60 met the criteria for inclusion in the study. Three patients were excluded because of immediate transfer after treatment (n = 1) or unclear details concerning outcomes in the medical records (n = 2). Fourteen of the 60 patients underwent CO_2_ angiography after no active bleeding was observed during ICM angiography and seven underwent CO_2_ angiography because the target vessel was unclear owing to spontaneous cessation of active bleeding, giving a total of 21 patients. Eight further patients underwent CO_2_ angiography for confirmation of hemostasis after embolization. Finally, there were 29 patients in the CO_2_ angiography group and 31 in the ICM angiography group (Fig. 2).

The baseline characteristics of the study participants are summarized in Table 1. Twenty-three percent of the patients were taking anticoagulants and 25% were on antiplatelet agents. Coagulopathy was identified in 28% of the patients and hemodynamic instability in 40%. Eighty-five percent of the bleeding episodes originated from the right side of the colon. Of the 60 included patients, 57 underwent contrast-enhanced CT before undergoing TAE. Among these 57 patients, active bleeding was detected in 48, leading to TAE. The three patients who did not undergo contrast-enhanced CT and the nine patients in whom active bleeding was not detected by contrast-enhanced CT were found to have active bleeding either through endoscopy or continued to have clinical signs of bleeding, leading to the decision to proceed with TAE.

**Table 1:** Baseline characteristics of the study population

Parameter	Total (*n* = 60)	ICM angiography (*n* = 31)	CO_2_ angiography (*n* = 29)
**Patient characteristics**			
Age (years) median [IQR]	74 [65, 80]	70 [62, 81]	74 [68, 80]
Male sex, n (%)	43 (72)	25 (81)	18 (62)
BMI (kg/m^2^), median [IQR]	22.1 [19.4, 24.5]	22.5 [20.8, 24.5]	21.3 [18.8, 23.5]
**Comorbidities, n (%)**			
Hypertension	29 (48)	16 (52)	13 (45)
Diabetes mellitus	18 (30)	12 (39)	6 (21)
Hemodialysis	6 (10)	3 (9.7)	3 (10)
**Medications, n (%)**			
Anticoagulants	14 (23.3)	7 (22.6)	7 (24)
Antiplatelet agents	15 (25)	9 (29)	6 (21)
NSAIDs	2 (3.3)	1 (3.2)	1 (3.4)
Steroids	8 (13)	4 (13)	4 (14)
**Clinical manifestations and laboratory tests**			
Hemoglobin, g/dL, median [IQR]	8.0 [7.0, 9.8]	8.0 [7.1, 9.7]	8.6 [7.0, 10.4]
eGFR (ml/min per 1.73 m^2^), median [IQR]	57.7 [43.8, 72.3]	65 [52, 73]	55 [29, 71]
BUN, mg/dl, median [IQR]	17.2 [11.1, 26.4]	16 [9.9, 22.7]	19.6 [12.2, 26.9]
Coagulopathy, n (%)	17 (28)	11 (36)	6 (21)
Platelet count (10^4^/μL), median [IQR]	17.1 [13.6, 21.3]	17.2 [14.3, 20.9]	17.0 [12.7, 21.6]
PT-INR, median [IQR]	1.15 [1.05, 1.39]	1.11 [1.02, 1.48]	1.15 [1.06, 1.33]
APTT, s, median [IQR]	33.4 [27.7, 37.4]	33.5 [27.4, 38.4]	30.9 [28.0, 37.3]
Hemodynamic instability, n (%)	24 (40)	12 (39)	12 (41)
**CT findings**			
Bleeding from the right colon, n (%)	51 (85)	27 (87)	24 (83)
Interval between CT and angiography, minutes, median [IQR]	81 [48, 144]	84 [49, 144]	84 [50, 148]

The IQR represents the 25th and 75th percentiles. *APTT* activated partial thromboplastin time, *BMI* body mass index, *BUN* blood urea nitrogen, *CO*_*2*_ carbon dioxide, *CT* computed tomography, *eGFR* estimated glomerular filtration rate, *ICM* iodinated contrast medium, *IQR* interquartile range, *NSAIDs* nonsteroidal anti-inflammatory drugs, *PT-INR* prothrombin time-international normalized ratio

Active bleeding was identified by ICM angiography in 42 of the 60 patients and not identified in 18. Bleeding ceased spontaneously in 11 of the 42 patients identified to have active bleeding, with no evidence of guidewire-induced spasm or thrombus formation during the angiographic procedure. Twenty-one patients underwent CO_2_ angiography to identify the bleeding source when no active bleeding was detected by ICM angiography, and active bleeding was detected in 10 (48%) of these patients by CO_2_ angiography. Twelve further patients underwent CO_2_ angiography post-embolization, four (33%) of whom were found to have active bleeding by CO_2_ angiography. There were some complications in the CO_2_ angiography group, including one case of abdominal pain and another of vomiting, both of which were transient and did not require treatment. Embolization was possible in 50 of the 60 cases, resulting in a technical success rate of 83%. Technical failure occurred in one case as a result of vasospasm, which led to disappearance of active bleeding and the target vessel becoming unclear, preventing embolization. In nine cases, embolization could not be performed because of absence of active bleeding and the target vessel being unclear. Further details of the embolization procedures are shown in Table 2. Embolization was mostly performed on a single vasa recta, with microcoils being the most commonly used embolic material, followed by imipenem/cilastatin.

**Table 2:** Details of embolization and clinical outcomes in patients undergoing transcatheter arterial embolization

Parameter	Overall (*n* = 50)	ICM angiography (*n* = 28)	CO_2_ angiography (*n* = 22)
**Embolization details, n (%)**			
Embolized vasa recta			
1	32 (64)	17 (61)	15 (68)
2	4 (8)	2 (7.1)	2 (9.1)
> 2	14 (28)	9 (32)	5 (23)
Empirical embolization	6 (12)	4 (14)	2 (9.1)
Embolic method			
Microcoils alone	28 (56)	14 (50)	14 (64)
IPM/CS alone	12 (24)	7 (25)	5 (23)
Microcoils and IPM/CS	5 (10)	4 (14)	1 (4.5)
NBCA	2 (4)	1 (3.6)	1 (4.5)
Microcoils, IPM/CS and GS	1 (2)	1 (3.6)	0 (0)
IPM/CS and NBCA	1 (2)	1 (3.6)	0 (0)
GS	1 (2)	0 (0)	1 (4.5)
**Clinical outcomes, n (%)**			
Major ischemic complication	3 (6)	2 (7.1)	1 (4.5)
Rebleeding	6 (12)	5 (18)	1 (4.5)

*CS* cilastatin, *CO*_*2*_ carbon dioxide, *GS* gelatin sponge, *ICM* iodinated contrast medium, *IPM* imipenem, *NBCA* N-butyl cyanoacrylate, *TAE* transcatheter arterial embolization

The treatments and clinical outcomes are summarized in Table 3. Nine out of 60 patients experienced rebleeding during hospitalization. The rebleeding rate was higher in the ICM group than in the CO_2_ angiography group (23% vs. 6.9%). Among the patients who underwent TAE, the rebleeding rate was 18% in the ICM angiography group and 5% in the CO_2_ angiography group. The overall rate of major ischemic complications was 6%; the between-group difference was not statistically significant (ICM angiography group, 7.1%; CO_2_ angiography, 4.5%). Two major ischemic complications required surgery for gastrointestinal necrosis and one of ischemic abdominal pain was treated by fasting. The median hospital stay was similar between the ICM angiography and CO_2_ angiography groups (9 days vs. 8 days); the in-hospital mortality was also comparable (3.2% vs. 3.4%), with the causes of death being unrelated to lower gastrointestinal bleeding or the effects of embolization.

**Table 3:** Descriptive statistics for outcome measures

Parameter	Overall (*n* = 60)	ICM angiography (*n* = 31)	CO_2_ angiography (*n* = 29)
**Treatment**			
Massive transfusion	25 (42)	14 (45)	11 (38)
PRBC (ml), median [IQR]	1200 [600, 2100]	1200 [900, 2100]	900 [300, 1800]
FFP (ml), median [IQR]	0 [0, 720]	0 [0, 700]	0 [0, 720]
TAE, n (%)	50 (83)	28 (90)	22 (76)
**Clinical outcomes**			
Rebleeding, n (%)	9 (15)	7 (23)	2 (6.9)
Length of hospital stay (days), median [IQR]	9 [6, 14]	9 [7, 18]	8 [6, 14]
In-hospital mortality, n (%)	2 (3.3)	1 (3.2)	1 (3.4)

The IQR represents the 25th and 75th percentiles. *CO*_*2*_ carbon dioxide, *FFP* fresh frozen plasma, *ICM* iodinated contrast medium, *IQR* interquartile range, *PLT* platelets, *PRBC* packed red blood cells

## Discussion

The primary focus of this study was on the potential of CO_2_ angiography to reduce the risk of repeat bleeds in patients with diverticular bleeding. The rebleeding rate tended to be lower in the CO_2_ angiography group than in the ICM angiography group. Furthermore, CO_2_ angiography demonstrated an ability to detect bleeding sources that were not evident on ICM angiography, indicating its usefulness in the diagnosis and treatment of diverticular bleeding. This finding suggests that CO_2_ angiography can facilitate more accurate identification of bleeding sources and enable TAE, potentially reducing the risk of rebleeding. To the best of our knowledge, this study provides the first detailed analysis of clinical outcomes using CO_2_ angiography, and its results are expected to contribute to improved diagnostic and treatment approaches in clinical practice.

In this study, the rate of detection of active bleeding was 47.6% using CO_2_ angiography and 33.3% using ICM angiography after embolization procedures. Although our detection rate using CO_2_ angiography is lower than the 57% reported previously [11], we found that CO_2_ angiography could reveal bleeding sources that were not identified by ICM angiography. The difference in detection rates between the studies may reflect variations in patient populations and sample sizes. The detection rate was within the range reported for provocative angiography using urokinase or heparin (30%–50%) [12, 17, 18]. Provocative angiography may also promote bleeding at puncture sites and other bleeding not related to lower gastrointestinal hemorrhage [12, 19]. Complications from CO_2_ angiography in the lower gastrointestinal tract were minor and transient, confirming the safety of this procedure. These findings indicate that CO_2_ angiography is useful for diagnosis of active bleeding in cases of diverticular hemorrhage. CO_2_ angiography may also be a safe alternative for patients with renal dysfunction or allergies to ICM, so has the potential for broader application in diagnosing and treating lower gastrointestinal bleeding.

Our rebleeding rate in the CO_2_ angiography group was 6.9%, which is significantly lower than the previously reported rebleeding rates of 17%–28% after TAE and the rate of 53% when embolization could not be performed owing to an inability to confirm active bleeding [10]. This could be attributed to the vasodilatory effect and low viscosity of CO_2_. Typically, vasoconstriction and thrombus formation play a role in hemostasis during bleeding [20]; however, owing to its properties, CO_2_ is thought to dilate these constricted vessels and leak from thin vessel branches and thrombi, potentially promoting bleeding. By inducing bleeding, embolization can be performed, potentially reducing the risk of rebleeding.

The frequency of major ischemic complications in this study was not significantly different from that previously reported [6, 8, 9], although there have been concerns about an increase in adverse events after embolization. This result may be attributed to the predominant embolic material used in our study, which was microcoils. When embolizing the vasa recta with microcoils, proximal embolization occurs, which is considered to lower the risk of ischemic complications [7]. Furthermore, in this study, embolization was often performed when the vasa recta consisted of two or fewer vessels, with super-selective embolization as the primary approach. This may have contributed to minimizing ischemic events [7]. Additionally, CO_2_ angiography was used to induce active bleeding, which allowed targeting of only the specific vessels responsible for the hemorrhage; thereby, reducing the extent of ischemia to the bowel and enhancing the overall safety of the procedure.

These results indicate that CO_2_ angiography could be an effective option for management of the risk of rebleeding and have significant implications for treatment strategies in patients with diverticular bleeding.

This study had several limitations. First, it had a retrospective design, which means that the possibility of bias cannot be excluded. Second, the small sample size may limit the generalizability of the results and the statistical power of the analysis. Third, the lack of consistency in choice of embolic material and variations in the application of CO_2_ angiography by different operators could have affected treatment outcomes. Moreover, as noted in previous research [21], lower gastrointestinal bleeding is intermittent, which means that the timing of examination could have significantly affected the rate of detection by CO_2_ angiography. Therefore, it is not possible to completely exclude the impact of timing on the detection rate. A randomized controlled study is necessary to address this problem.

## Conclusion

This study demonstrated the potential of CO_2_ angiography to reveal the site of active bleeding in cases of diverticular bleeding that are not detectable on ICM angiography. The trend toward lower rebleeding rates in the CO_2_ angiography group suggests that CO_2_ angiography could be an effective option for the management of diverticular bleeding.

## Data Availability

The data associated with this research are available from the corresponding author upon reasonable request.

## Abbreviations

CO_2_: Carbon dioxide
ICM: Iodinated contrast medium
TAE: Transcatheter arterial embolization
CT: Computed tomography
SMA: Superior mesenteric artery
IQR: Interquartile range
